# Inflammatory Milieu and Specific T-Cell Response Observed Three Months and One Year After SARS-CoV-2 Infection in Long COVID Subjects

**DOI:** 10.3390/ijms262110412

**Published:** 2025-10-27

**Authors:** Eleonora Cimini, Alessandra Vergori, Claudia Cimaglia, Eleonora Tartaglia, Stefania Notari, Francesca Colavita, Giulia Matusali, Ilaria Mastrorosa, Valentina Mazzotta, Pierangelo Chinello, Paola Mencarini, Maria Letizia Giancola, Amina Abdeddaim, Rita Casetti, Germana Grassi, Simona Gili, Flavia Cristofanelli, Fabrizio Maggi, Pierluca Piselli, Enrico Girardi, Andrea Antinori, Marta Camici

**Affiliations:** 1 Laboratory of Cellular Immunology and Pharmacology, National Institute for Infectious Diseases “Lazzaro Spallanzani” IRCCS, 00149 Rome, Italy; 2 HIV/AIDS Unit, National Institute for Infectious Diseases “Lazzaro Spallanzani” IRCCS, 00149 Rome, Italy; 3 Epidemiology Unit, National Institute for Infectious Diseases “Lazzaro Spallanzani” IRCCS, 00149 Rome, Italy; 4 Laboratory of Virology and Biosafety Laboratories, National Institute for Infectious Diseases “Lazzaro Spallanzani” IRCCS, 00149 Rome, Italy; 5 Clinical Department, National Institute for Infectious Diseases “Lazzaro Spallanzani” IRCCS, 00149 Rome, Italy; 6 Scientific Direction, National Institute for Infectious Diseases “Lazzaro Spallanzani” IRCCS, 00149 Rome, Italy

**Keywords:** SARS-CoV-2, inflammation, coagulation, T-cell response, long COVID, cytokines

## Abstract

Long COVID (LC) is characterized by a wide range of symptoms, the causes of which remain unclear. We investigated associations between inflammatory and coagulation factors, adaptive immune response to SARS-CoV-2, and LC. We enrolled 196 unvaccinated individuals with SARS-CoV-2 (March–June 2020). Blood samples were collected at three (T3M) and twelve (T12M) months post infection. Plasma concentrations of coagulation factors (D-Dimer, E-Selectin, ICAM-1, VCAM-1) and inflammatory markers (IL-6, IL-8, TNF-α, IL-1β) were measured by ELISA, and SARS-CoV-2-specific T-cell response was assessed by Elispot. LC occurred in 66/196 patients (34%); 77.8% had been hospitalized. Respiratory symptoms were present in 54%, fatigue in 30%, and neuropsychological symptoms in 14%. At T3M, hospitalized patients exhibited higher levels of ICAM-1, VCAM-1, and IL-6, along with increased immunoreactivity. LC patients exhibited elevated IL-8 and TNF-α and enhanced immunoreactivity at T3M, though these differences were not observed at T12M. Inflammatory and coagulation markers were altered at three months after acute infection, with some changes persisting at one year, suggesting a long-term immunological impact of SARS-CoV-2 on the inflammatory response. A SARS-CoV-2-specific T-cell response was still detectable at T12M, albeit at a lower level than at T3M, suggesting the persistence of protective memory T-cells beyond the acute phase.

## 1. Introduction

Post-COVID-19 condition, commonly referred to as long COVID (LC), is now internationally recognized, along with other terms describing prolonged or emerging symptoms following SARS-CoV-2 infection. LC is generally defined by the persistence or onset of new symptoms at least three months after the initial infection [1].

It is estimated that approximately 45% of COVID-19 survivors, regardless of hospitalization status, experience a range of persistent symptoms around four months post infection. However, current understanding is limited due to heterogeneity in study designs, follow-up durations, and measurement methods [2].

LC encompasses a wide spectrum of debilitating symptoms affecting multiple organ systems, including cardiopulmonary complications, relentless fatigue, and neurocognitive dysfunction, which can significantly impair daily functioning and quality of life [1]. Managing this condition remains a significant challenge due to limited understanding of its pathophysiological mechanisms. Addressing this critical health issue is essential for improving patient outcomes and developing effective treatment strategies [1,3,4,5,6,7,8].

One of the key mechanisms implicated in LC is endothelial dysfunction, widely recognized as a central pathological feature of COVID-19 and its long-term consequences [9,10,11]. Patients suffering from long-lasting symptoms often exhibit higher levels of pro-inflammatory molecules in plasma, such as tumor necrosis factor-alpha (TNF-α), interferon-gamma (IFN-γ), interleukin-2 (IL-2), and interleukin-6 (IL-6) [10,11]. The ongoing immune activation, driven by sustained inflammatory stimuli, may activate coagulation pathways, contributing to the persistence of symptoms and the occurrence of cardiovascular events in these patients [3,12]. Furthermore, several studies have shown that individuals experiencing LC often face ongoing immune dysregulation [13,14,15,16].

The findings indicate an enhanced immune response involving CD4+ and CD8+ T-cells that are specific to SARS-CoV-2, along with increased antibody affinity [17]. These findings suggest that the symptoms of LC may, at least in part, stem from chronic immune activation, possibly caused by the persistent presence of SARS-CoV-2 antigens in the body.

To further elucidate these mechanisms, this study aims to characterize inflammatory and coagulative profiles, as well as the SARS-CoV–2–specific T-cell response, in unvaccinated individuals referred to our institute during the first wave of the COVID-19 pandemic, up to 12 months after acute infection, regardless of the presence of long COVID syndrome.

Coagulation and endothelial markers D-dimer, E-selectin (E-Sel), Intercellular Adhesion Molecule-1 (ICAM-1), and Vascular Cell Adhesion Molecule-1 (VCAM-1), together with key inflammatory cytokines (IL-6, IL-8, TNF-α, IFN-γ, IL-1β), will be assessed at three and twelve months postSARS-CoV-2 infection. The secondary objectives are to determine whether the levels of these biomarkers differ between patients with chronic symptoms (long COVID) and those who are asymptomatic post-COVID, and to explore potential correlations between changes in biomarker concentrations and long COVID symptoms over time.

We hypothesize that long COVID symptoms are associated with the complex interplay between inflammation, coagulation, and immune response at three and twelve months following SARS-CoV-2 infection. By correlating these biological markers with clinical manifestations, we aim to identify mechanistic pathways that may improve the understanding and management of long COVID in an unvaccinated cohort.

## 2. Results

### 2.1. Study Population

The clinical and demographic characteristics of the study population are presented in Table 1. Of the 196 participants, 119 (60.7%) were male and 77 (39.3%) female. The median age was 56.5 years (interquartile range [IQR], 49.5–64.9). The median time between the first reported positive SARS-CoV-2 test and the baseline ambulatory follow-up visit was 86 days (interquartile range, 78–91 days). All participants were experiencing their first episode of COVID-19.

Among the participants, 147 (75%) had previously been hospitalized for COVID-19 at our institute, while 18.9% had not required hospitalization. A smaller proportion had been hospitalized either at another facility before being transferred to INMI (3.6%) or exclusively at another center (2.6%). The median interval between hospital discharge and follow-up was 82 days (interquartile range, 76–89), with a median hospitalization duration of 11 days (interquartile range, 8–18). Only a minority of hospitalized patients (2.0%) required admission to the intensive care unit (ICU).

Regarding comorbidities, 11.7% of patients reported a history of chronic respiratory disease, while 32.7% did not. However, detailed information on pre-existing conditions was not consistently available, as the clinical implications of long COVID were not yet fully recognized at that time. To partially address this limitation, we inferred the presence of chronic conditions indirectly by examining ongoing therapies at the first follow-up. The most commonly reported medications were bisoprolol (5%), pantoprazole (4.6%), ramipril (4.1%), low-dose acetylsalicylic acid (3.5%), and amlodipine (3.5%). This pattern reflects the high prevalence of cardiovascular comorbidities in the cohort, with frequent use of beta-blockers, ACE inhibitors, antiplatelet agents, and calcium channel blockers. Nevertheless, this approach does not clarify whether these therapies—and the underlying conditions—were pre-existing or initiated as a consequence of COVID-19 or its complications.

At the first follow-up visit, 66 patients (33.7%) reported at least one symptom attributable to long COVID. Of these, 45 (68.2%) reported a single symptom, while 21 (31.8%) reported multiple symptoms (14 with two, five with three, and two with four). A total of 96 symptoms were recorded across all symptomatic patients. The most frequently reported were dyspnea, cough, or shortness of breath (54.5% of symptomatic patients; 18.4% of the total cohort), followed by fatigue (30.3%; 10.2%), other symptoms (24.2%; 8.2%), neuropsychological symptoms (13.6%; 4.6%), anosmia/ageusia/dysosmia/dysgeusia (12.1%; 4.1%), and arthromyalgias (10.6%; 3.6%) (Table 1).

At the 12-month follow-up, 48 patients completed their evaluation only with laboratory tests and did not report significant symptoms. These participants were subsequently contacted to continue follow-up through blood sampling for immunological analyses.

### 2.2. Biological Differences Between Asymptomatic and LC Patients

**Figure 1:**
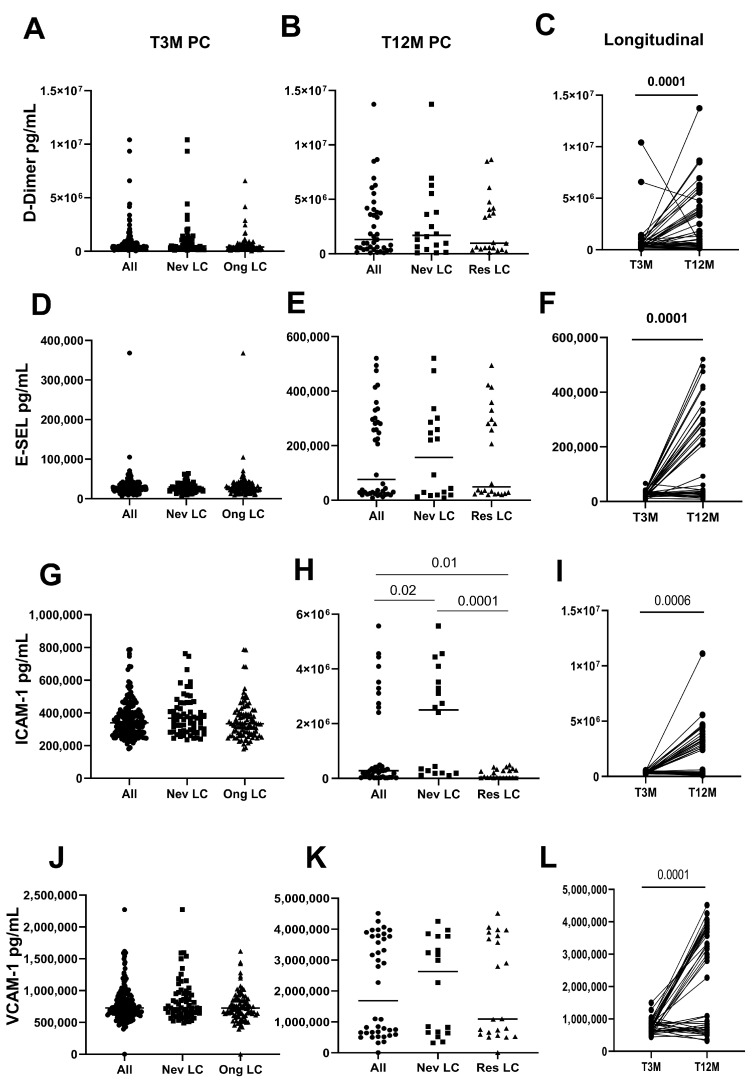
Coagulative and endothelial factors after three and twelve months post SARS-CoV-2 infection according to the onset of symptoms. Plasmatic concentrations of D-Dimer (**A**–**C**), E-Sel (**D**–**F**), ICAM-1 (**G**–**I**), and VCAM-1 (**J**–**L**) were measured in 168 (All, Black circles) LC subjects (Never LC: 68, Black squares; Ongoing LC: 100, Black triangles) at three and twelve months (All: 40; Never LC: 18; Resolved LC: 22) post SARS-CoV-2 infection using the ELISA test.

Regarding the coagulation profile, at T3M, no differences were observed in the levels of D-Dimer (Figure 1A–C), E-Selectin (Figure 1D–F), ICAM-1 (Figure 1G–I), and VCAM-1 (Figure 1J–L) between asymptomatic (Never LC) and symptomatic LC (Ongoing LC) subjects. In contrast, we found that levels of ICAM-1 (*p* = 0.006, Figure 1G) and VCAM-1 (*p* = 0.02, Figure 1J) were higher in hospitalized subjects compared to their non-hospitalized counterparts, indicating greater endothelial activation (Supplementary Figure S1). At T12M, 48 of the 196 subjects returned for their annual check-up. When comparing values from T3M to T12M within the available data, we found that levels of ICAM-1 (*p* < 0.0001) were higher in Never LC subjects at T12M compared to those in Resolved LC (Figure 1H). No differences were observed for the other factors between Never LC and Ongoing LC subjects. Moreover, analyzing the data in a longitudinal course, we observed a significant increase in D-Dimer, E-Selectin, and VCAM-1 (*p* < 0.0001 for all, Figure 1C,F,L), along with a substantial rise in ICAM-1 (*p* < 0.0006, Figure 1I), suggesting progressive endothelial involvement over time.

**Figure 2:**
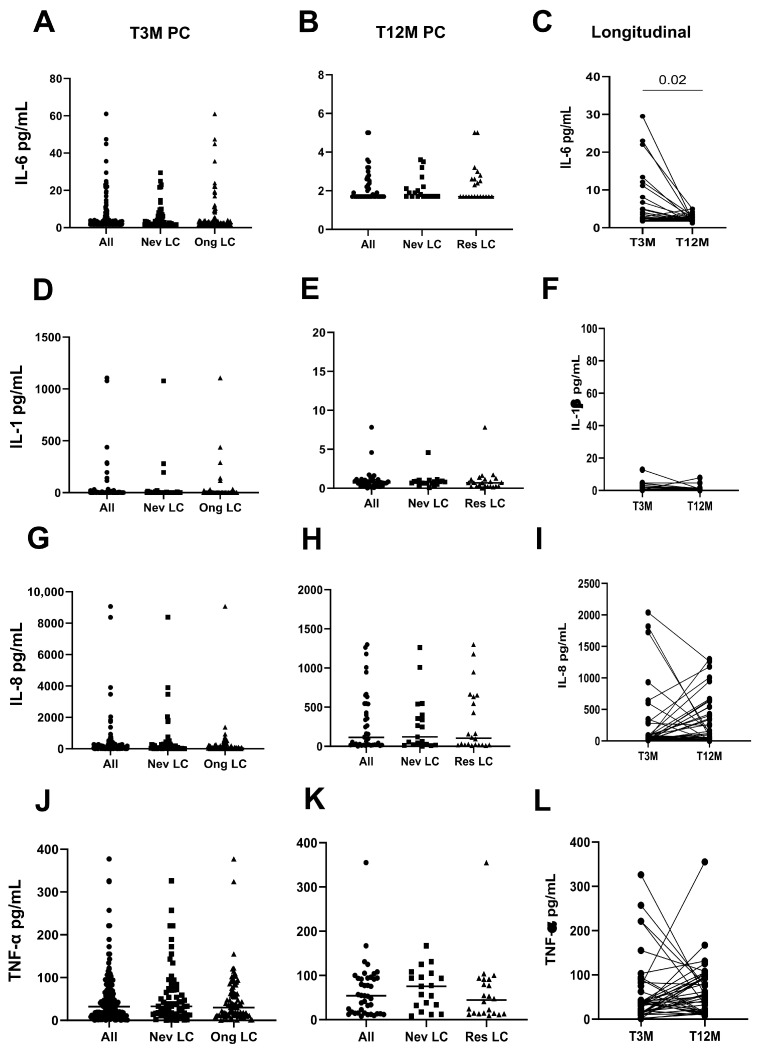
Inflammatory factors after three and twelve months post SARS-CoV-2 infection, according to symptoms’ onset. Plasmatic concentrations of inflammatory factors such as IL-6 (**A**–**C**), IL-1β (**D**–**F**), IL-8 (**G**–**I**), and TNF-α (**J**–**L**) were measured in 192 LC subjects (Never LC, 71; Ongoing LC: 118) at three and twelve months [All: 41 (Black circles); Never LC: 19 (Black squares); Resolved LC: 22 (Black triangles)] post SARS-CoV-2 infection using the ELISA test.

The inflammatory profile revealed that no differences were observed for all cytokines in Never LC and Ongoing LC/Resolved LC subjects at T3M (Figure 2A,D,G,J) and T12M (Figure 2B,E,H,K). In contrast, the analysis of the inflammatory profile at T3M revealed higher levels of IL-6 in hospitalized patients compared to non-hospitalized (*p* = 0.01, Supplementary Figure S2A). No group differences were observed between groups for IL-1β (*p* = 0.51, Supplementary Figure S2), IL-8 (*p* = 0.11, Supplementary Figure S2), and TNF-α (*p* = 0.36, Supplementary Figure S2).

When comparing T3M with the T12M mark, IL-6 levels experienced a significant decline over time (*p* = 0.02, Figure 2C), while IL-1β (*p* = 0.30, Figure 2F), IL-8 (*p* = 0.17, Figure 2I), and TNF-α (*p* = 0.13, Figure 2L) showed no notable changes.

**Figure 3:**
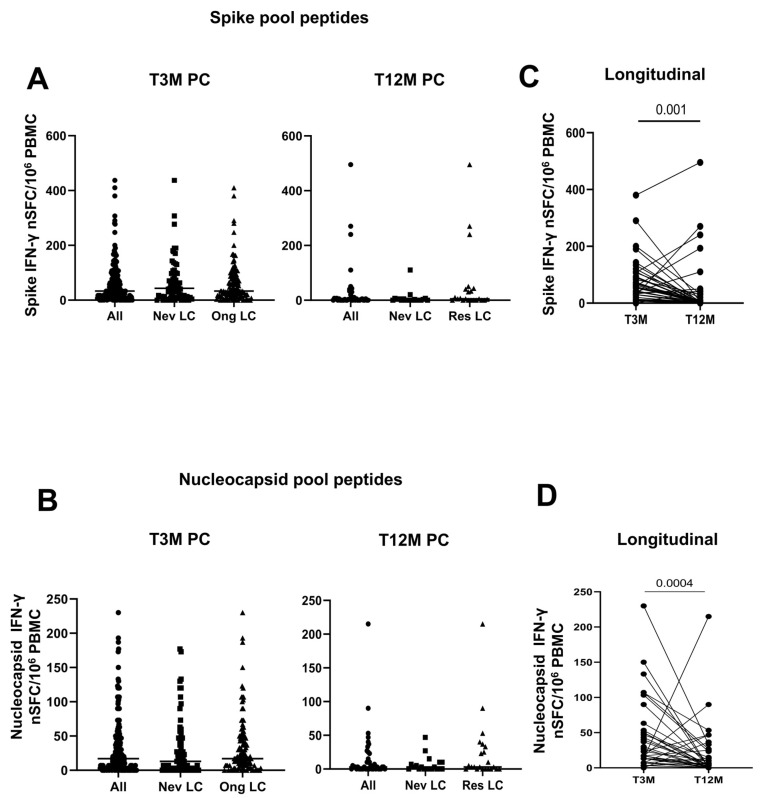
T-cell response to SARS-CoV-2 at three and twelve months post infection, according to symptoms’ onset. Spike- (**A**,**C**) and nucleocapsid (**B**,**D**)-specific T-cell responses were measured in PBMC from 189 (All, Black circles) [Never LC, 71 (Black squares); Ongoing LC: 118 (Black triangles)] LC subjects at three and twelve months (All: 41; Never LC: 19; Resolved LC: 22) post SARS-CoV-2 infection by Elispot assay.

Lastly, our data showed that there is no difference in the strength of the spike (Figure 3A,C) and nucleocapsid T (Figure 3B,D) response between Never LC and Ongoing LC at T3M and Resolved LC at T12M. At the same time, we demonstrated that the spike–SARS-CoV-2 specific T-cell response was significantly higher in hospitalized patients compared to non-hospitalized patients (*p* = 0.004, Supplementary Figure S3A) and also declined markedly at the 12-month mark (T12M) in the hospitalized patient’s group (*p* = 0.0001, Supplementary Figure S3A). Overall, a significant reduction in spike-specific T-cell response was observed across the cohort (T12M, *p* = 0.001, Figure 3C and Supplementary Figure S3C).

In addition, the nucleocapsid-specific T-cell response maintained a comparable strength between hospitalized and non-hospitalized subjects at T3M (*p* = 0.34, Supplementary Figure S3B) and similarly experienced a significant reduction by T12M (*p* = 0.0001, Figure 3D). The longitudinal comparisons between T3M and T12M confirm the waning of the adaptive immune response one year after the acute infection (*p* = 0.0004, Figure 3D and Supplementary Figure S3D).

### 2.3. Biological Differences Between Patients with and Without LC According to Gender Classification

**Figure 4:**
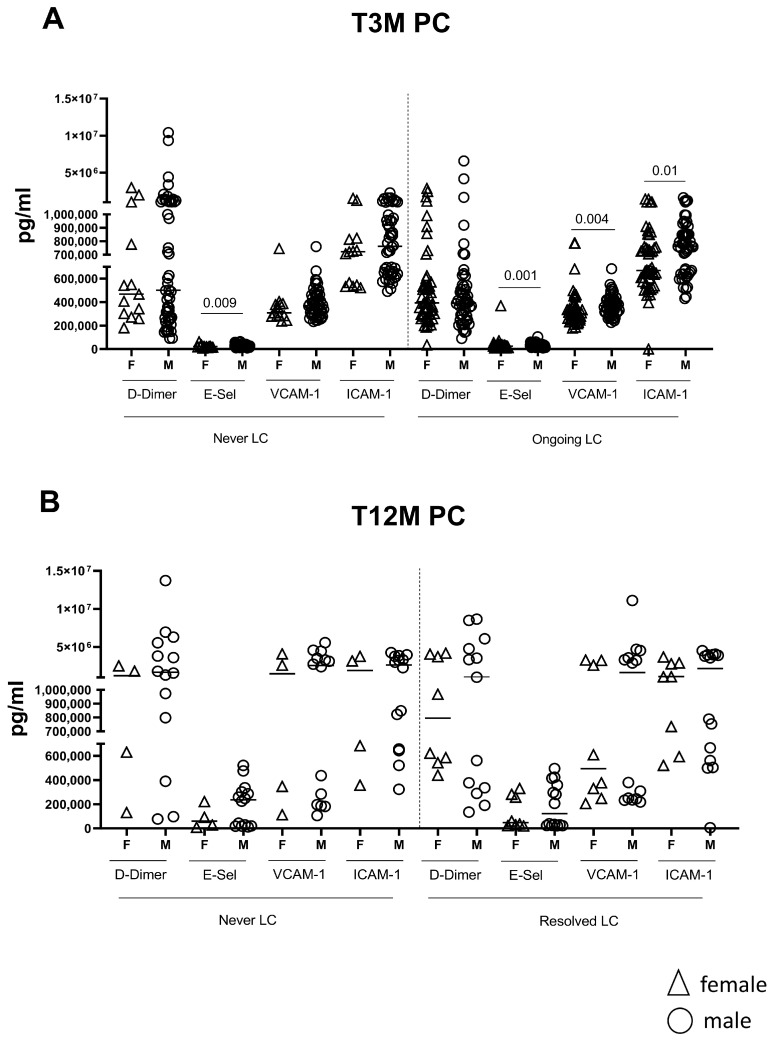
Coagulative and endothelial factors after three and twelve months post SARS-CoV-2 infection, according to gender classification. Plasmatic concentrations of D-Dimer, E-Sel, ICAM-1, and VCAM-1 were measured in 168 (Never LC: 13 Female and 55 Male; Ongoing LC: 50 Female and 50 Male) LC subjects at three (**A**) and twelve months (**B**) (Never LC: 4 Female and 14 Male; Resolved LC: 8 Female and 13 Male) post SARS-CoV-2 infection using the ELISA test, according to the presence or not of symptoms and according to gender analysis.

At T3M post SARS-CoV-2 infection, we found no significant differences in coagulation or endothelial markers between patients with LC and Ongoing LC and non-LC (Never LC) participants, regardless of sex. Specifically, D-Dimer, E-selectin, and VCAM-1 levels were similar among Ongoing LC and Never LC in both females and males (all *p* > 0.1; Figure 4A). However, within the LC group, male patients (n = 50) exhibited significantly higher expression of several crucial endothelial factors compared to female patients (n = 13): E-Sel (*p* = 0.001), VCAM-1 (*p* = 0.004), and ICAM-1 (*p* = 0.01, Figure 4A); D-Dimer expression remained comparable between LC females and males (*p* = 0.61, Figure 4A). However, these differences did not persist at T12M (Figure 4B).

**Figure 5:**
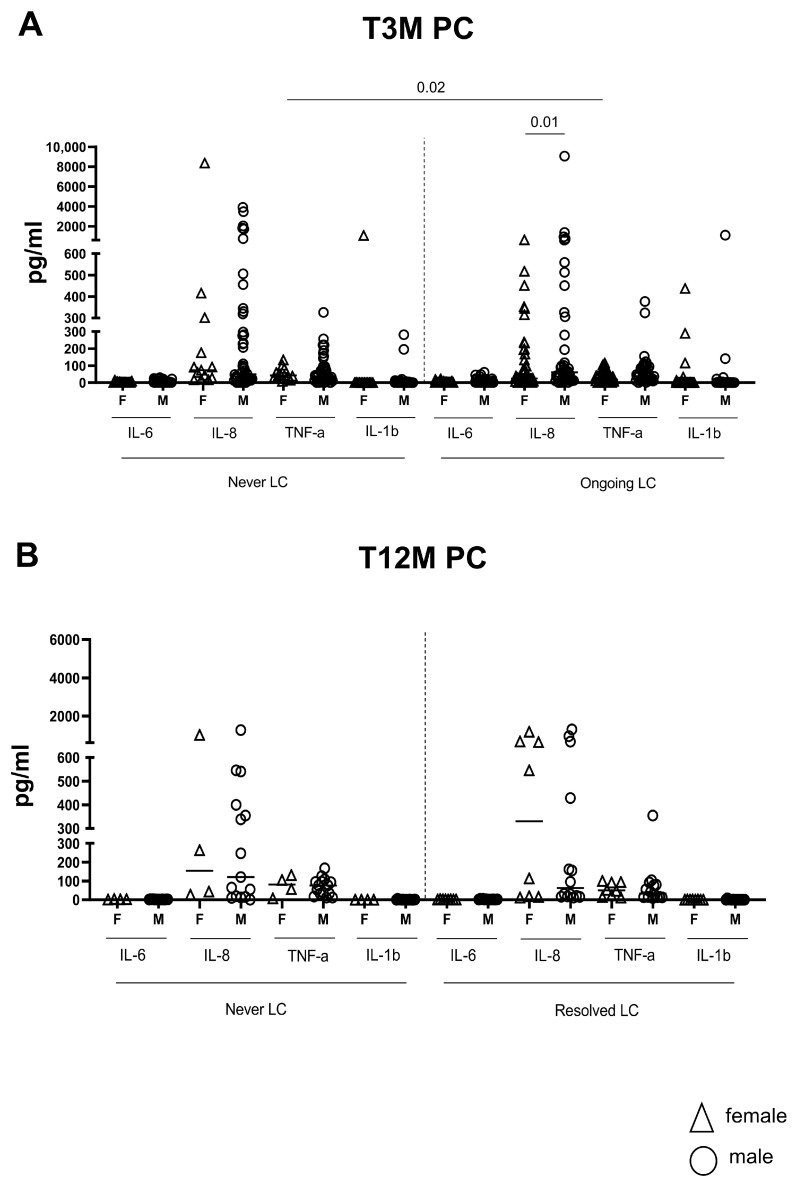
Inflammatory factors after three and twelve months post SARS-CoV-2 infection, according to gender classification. Plasmatic concentrations of inflammatory factors such as IL-6, IL-1, IL-8, and TNF-α were measured in LC 168 (Never LC: 16 Female and 55 Male; Ongoing LC: 50 Female and 50 Male) subjects at three (**A**) and twelve months (**B**) (Never LC: 4 Female and 14 Male; Resolved LC: 8 Female and 13 Male) post SARS-CoV-2 infection using the ELISA test, according to the presence or not of symptoms and according to gender analysis.

The inflammatory profile was strikingly characterized by elevated TNF-α levels in female patients with Ongoing LC (N = 49) compared to their Never LC counterparts (N = 13; *p* = 0.02, Figure 5A). In contrast, LC males showed significantly higher IL-8 levels compared with LC females (*p* = 0.01, Figure 5A). Other inflammatory factors did not display significant differences: IL-6 levels were similar for LC and non-LC females (*p* = 0.60) and males (*p* = 0.68); IL-8 levels were comparable for LC and non-LC females (*p* = 0.06) and males (*p* = 0.88); along with TNF-α levels in males (*p* = 0.55) and IL-1β levels for both females (*p* = 0.79) and males (*p* = 0.42). Crucially, all these differences were resolved at T12M (Figure 5B).

**Figure 6:**
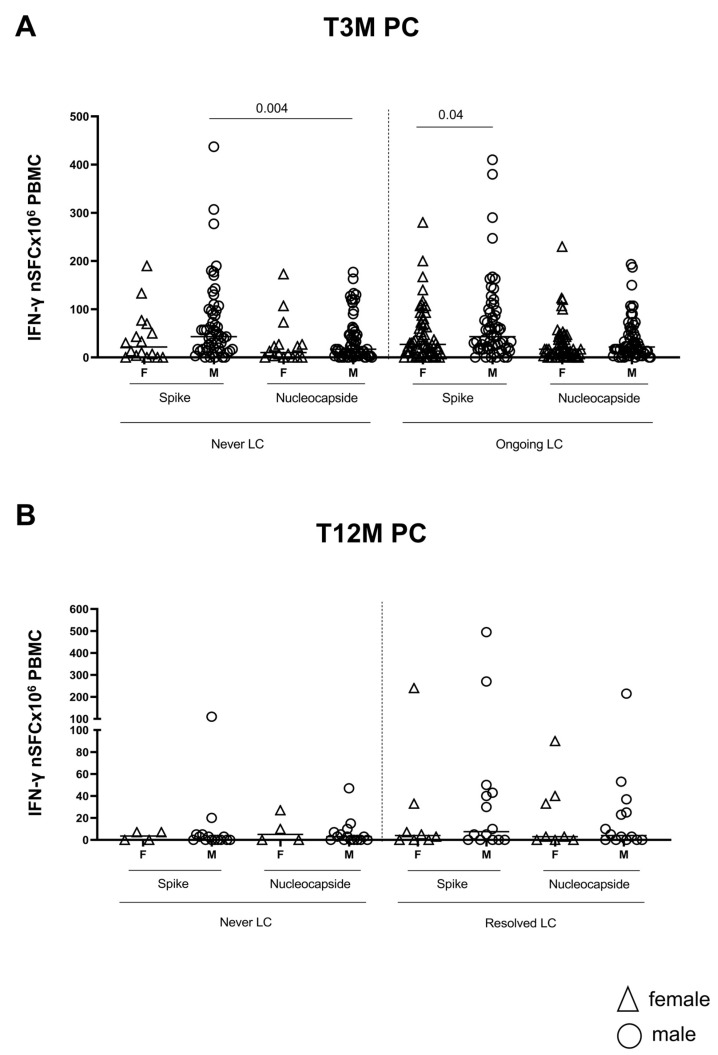
T-cell response to SARS-CoV-2 after three and twelve months post infection, according to gender classification. Spike and nucleocapsid-specific T-cell responses were measured in PBMC from 168 (Never LC: 16 Female and 55 Male; Ongoing LC: 50 Female and 50 Male) LC subjects at three (**A**) and twelve months (**B**) (Never LC: 4 Female and 14 Male; Resolved LC: 8 Female and 13 Male) post SARS-CoV-2 infection by Elispot assay, dividing the LC subjects for the absence of symptom and according to gender analysis.

Regarding adaptive immunity, the spike-specific T-cell response was similar between Ongoing LC and Never LC female patients (spike Ongoing LC vs. Never LC females = 0.52) and male patients (spike Ongoing LC vs. Never LC males = 0.88). However, a significantly stronger spike response emerged in male LC patients when compared to females (*p* = 0.04), underscoring the gender differences in immune response. Additionally, within the Never LC male group, the spike T-cell response outperformed the nucleocapsid response (*p* = 0.004). Notably, there were no significant differences between Ongoing LC and Never LC patients in the nucleocapsid T-cell response (Ongoing LC vs. Never LC females *p* = 0.53, and males *p* = 0.56; Figure 6A). Likewise, these differences were not observed at T12M (Figure 6B).

### 2.4. Heat Map Analysis

To investigate the long-term impact of SARS-CoV-2 on coagulation, inflammation, and adaptive T-cell response—and how these may vary with age—we performed a comprehensive heat map analysis utilizing Spearman’s correlation at T3M and T12M post infection. The aim was to identify markers mostly associated with long COVID (LC) and how these associations evolve.

At T3M, several relationships emerged among coagulation, endothelial, inflammatory, and immune markers. Age was positively correlated with E-Sel (R: 0.33, *p* = 0.03), VCAM-1 (R: 0.37, *p* = 0.01), and IL-1β (R: 0.30, *p* = 0.05), suggesting that endothelial activation and inflammation increase with age early in the post-COVID period. Diving deeper into the relationships among coagulative and endothelial factors, D-Dimer was significantly associated with VCAM-1 (R: 0.32, *p* = 0.03) while E-Sel demonstrated strong correlation with ICAM-1 (R: 0.62, *p* < 0.0001) and with VCAM-1 (R: 0.51, *p* < 0.00012 and an inverse correlation with IL-6 (R: −0.32, *p* = 0.03)

Examining the inflammatory network, IL-6 correlated with IL-1β (R: 0.39, *p* = 0.01), which in turn was strongly linked to IL-8 (R: 0.53, *p* < 0.0001) and TNF-α (R: 0.43, *p* = 0.004). In a striking display of interaction, IL-8 showed a robust positive correlation with TNF-α (R: 0.89, *p* < 0.0001), highlighting a potential co-regulated inflammatory axis.

**Figure 7:**
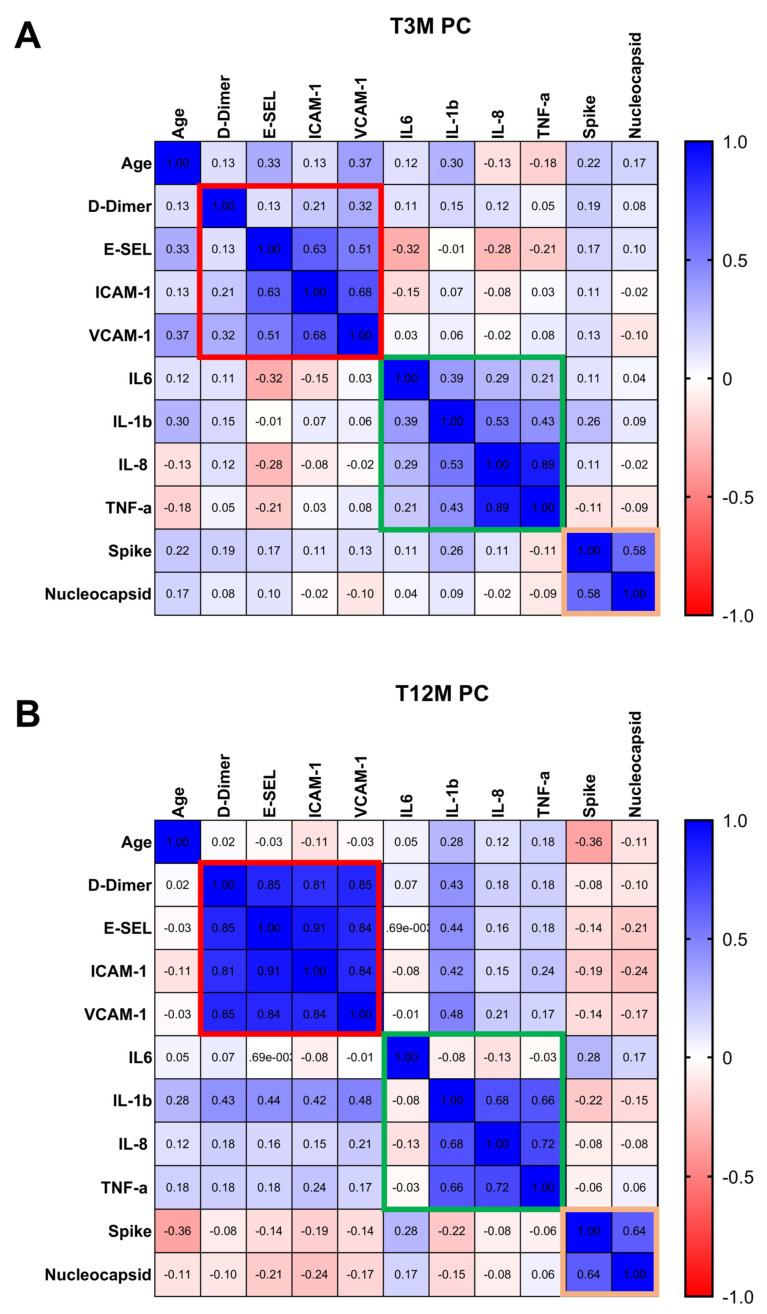
Heat map analysis of coagulative, inflammatory, and T-cell responses after three and twelve months post SARS-CoV-2 infection in LC subjects. The heatmap analysis depicts the correlation between all variables investigated in LC subjects at three (**A**) and twelve (**B**) months post SARS-CoV-2 infection. Brighter colors (e.g., red) indicate a stronger negative correlation, while darker colors (e.g., blue) indicate a stronger positive correlation. The Spearman correlation test was used, and the red square identified the correlation in the coagulative pathways, the green one the correlation in the inflammatory soluble factors. Finally, the orange square identified the correlation between the spike and nucleocapsid T-cell response.

Notably, the spike-specific T-cell response was positively correlated with the nucleocapsid-specific T-cell response (R: 0.58, *p* < 0.0001), suggesting a coordinated adaptive immunity in the early post-infection phase. The coagulative and endothelial factors are highlighted in a red square, while the inflammatory factors are marked in green. The adaptive SARS-CoV-2 response is indicated with an orange square (Figure 7A).

At the T12M milestone, among the 48 out of 196 patients who returned for follow-up, we observed persistent and, in some cases, stronger correlations, indicating ongoing dysregulation.

(Figure 7B). These critical findings are evidenced by the pronounced presence of red, green, and orange squares when compared to values at T3M (Figure 7B). Delving deeper, age exhibited a significant inverse correlation with the spike-adaptive T-cell response (R: −0.36, *p* = 0.01), suggesting that adaptive immunity may wane more sharply with age over time. Coagulation and endothelial markers demonstrated robust correlations: D-Dimer was strongly associated with E-Sel (R: 0.85, *p* < 0.0001), as well as with ICAM-1 (R: 0.80, *p* < 0.0001) and VCAM-1 (R: 0.85, *p* < 0.0001).

Notably, E-Sel demonstrated a strong positive correlation with ICAM-1 (R: 0.91, *p* < 0.0001), VCAM-1 (R: 0.84, *p* < 0.0001and IL-1β (R: 0.44, *p* = 0.004). Similarly, ICAM-1 was closely associated with VCAM-1 (R: 0.84, *p* < 0.0001) and IL-1β (R: 0.42, *p* = 0.006), while VCAM-1 correlated with IL-1β (R: 0.48, *p* = 0.001).

Turning to the inflammatory factors, compelling correlations were identified: IL-1β was significantly correlated with IL-8 (R: 0.68, *p* < 0.0001) and TNF-α (R: 0.66, *p* < 0.0001), while IL-8 showed a strong correlation with TNF-α (R: 0.72, *p* < 0.0001). Moreover, TNF-α revealed noteworthy correlations with IL-1β (R: 0.66, *p* = 1.77 × 10^−6^). Importantly, the spike-specific T-cell response was significantly correlated with the nucleocapsid response (R: 0.64, *p* < 0.0001), indicating a persistent, albeit declining, adaptive immune coordination.

## 3. Discussion

### 3.1. Clinical and Immunological Features of Long COVID

Our study supports and expands current evidence on the clinical and biological complexity of long COVID syndrome. The prevalence of LC in our cohort is 34%, aligning with previous existing reports, with respiratory symptoms, fatigue, and neuropsychological complaints being the most frequent clinical manifestations, underscoring the multisystemic impact of this condition [2]. The high prevalence of respiratory symptoms in our cohort reflects the pre-vaccination era during which the study was conducted.

From an immunological perspective, the findings of our analysis provide relevant insights into the long-term immune and inflammatory responses observed in patients post SARS-CoV-2 infection.

At three months post infection, hospitalized patients exhibited elevated levels of ICAM-1, VCAM-1, and IL-6, alongside a detectable immunoreactivity in SARS-CoV-2-specific T-cell response. This finding may reflect the higher viral load observed in hospitalized patients compared with outpatients during acute infection, and the subsequent stronger inflammatory cascade that developed in these patients. Moreover, post-COVID patients displayed a persistent pro-inflammatory cytokine profile, lasting up to one year post infection, independently of the presence of LC symptoms.

Interestingly, while IL-6 levels declined after twelve months, other inflammatory markers remained stable, highlighting IL-6 as a potential biomarker for monitoring the clinical status of LC patients [17]. Elevated IL-6 levels have been consistently reported during the acute phase of SARS-CoV-2 infection and in patients with prolonged post-COVID symptoms [18]. Several studies have also shown a gradual decrease in IL-6 concentrations over time in patients recovering from COVID-19, suggesting its involvement in the resolution of systemic inflammation and its potential utility as a longitudinal biomarker of recovery [19].

### 3.2. Coagulation and Endothelial Dysfunction

Our data revealed a sustained hypercoagulable state and endothelial dysfunction after SARS-CoV-2 infection.

In our cohort, 78% of subjects were hospitalized, and their profile was characterized by the extension of dysfunctional endothelial (ICAM-1) and inflammatory soluble factors (IL-8, IL-6) [20] and IL-1β [21] over time. This hyperinflammatory state was accompanied by coagulative disturbances, including elevated D-Dimer, E-sel, ICAM-1, and VCAM-1, aligning with previous studies highlighting COVID-19-associated coagulopathy as a key factor contributing to LC sequelae [22].

Persistent thrombo-inflammation, marked by the release of pro-inflammatory cytokines [23], platelet activation [24], endothelial damage [25,26], and complement system engagement [25,26,27], was evident in our data and others, with sustained elevated coagulative factors up to one year post infection [28].

At three months post infection, high levels of D-Dimer, E-Sel, ICAM-1, and VCAM-1 were observed, according to a previous study reporting that, at 3 months after COVID-19, D-Dimer level was increased in 15% of the subjects who had recovered from COVID-19 [29,30]. Notably, the perturbation of these coagulative factors lasted over time until one year after infection in our cohort, and independently of the presence of symptoms. These findings are in line with other studies reporting similar data associated with prolonged inflammation [30]. A higher level of endothelial activation factors in males compared with females appears to be justified by the presence of more severe acute disease [31]. Interestingly, longitudinal data show that endothelial activation markers remain elevated, more so in males than in females, regardless of the presence of long COVID symptoms. This finding may, on one hand, contribute to the increased cardiovascular risk associated with SARS-CoV-2 infection and, on the other, suggest a sex-specific mechanism underlying long COVID, in which endothelitis could represent a predisposing factor, in line with our results.

### 3.3. Adaptive Immune Dynamics

The adaptive immunity showed dynamic changes over time: hospitalized patients had higher SARS-CoV-2-specific T-cell responses at three months, which declined by one year. These responses were notably influenced by the larger viral inoculum and patient age [32,33].

A previous study reported that patients with pulmonary post-acute sequelae of SARS-CoV-2 infection (PASC) exhibited significantly higher frequencies of IFN-γ- and TNF-α-producing SARS-CoV-2-specific T-cells in peripheral blood compared with individuals without PASC [34]. The same study also found that elevated circulating levels of inflammatory biomarkers (IL-6 and CRP) positively correlated with both long COVID and the frequency of SARS-CoV-2-specific T-cells [34]. This suggests that, in the pre-vaccination era, pulmonary long COVID can be described as the progression of inflammatory alveolar damage triggered during the cytokine cascade typical of acute infection. The marked immuno-inflammatory activation likely reflects a proxy for the high alveolar viral load that occurred during the acute phase.

Accordingly, in our cohort we observed a stronger IFN-γ response of spike- and nucleocapsid-specific T-cells in hospitalized subjects compared with non-hospitalized participants, still detectable three months post infection but declining by one year. This trend is consistent with previous reports describing similar decreases at three [34], four [35], and six months [36] after infection. Consistent with previous studies identifying elevated IL-1β, IL-6, and TNF plasma levels as a cytokine signature of long COVID, we found that only TNF-α was significantly higher in long COVID compared with asymptomatic females at T3M [17].

The heat map analysis further confirmed the persistent perturbations of inflammatory, coagulative, and adaptive immune responses beyond the SARS-CoV-2 acute phase [37]. Supporting these findings, studies looking at immune dysregulation in subjects with LC who had mild acute COVID-19 have found T-cell alterations [38], including exhausted T-cells, reduced CD4+ and CD8+ effector memory cell numbers [39], and elevated PD-1 expression on central memory cells, persisting for at least 13 months. Moreover, several reports have shown that the SARS-CoV-2-T response in LC individuals suggested an improper crosstalk between the cellular and humoral adaptive immunity, which can lead to immune dysregulation, inflammation, and clinical symptoms associated with this debilitating condition [40,41]. Recently, research identified a unique immunological signature associated with LC syndrome, characterized by a persistent dysregulation of adaptive immune response [42]. LC patients restored their innate immune profiles comparable to those of healthy and recovered individuals. Still, in contrast, they showed deficits in T-cell and memory B-cell populations, differentiating LC from full recovery [18,43].

In addition, our data showed a strong impact of gender on the spike response after three months from infection. In particular, the spike-specific response was higher in males compared with females, both in LC (*p* = 0.04) and in asymptomatic patients at T3M. These findings suggest that a stronger spike-specific T-cell response may confer protection against the development of long COVID and be associated with more effective viral clearance [42]. This could represent one of the mechanisms underlying the higher risk of developing long COVID observed in females [18,44].

Several hypotheses have been proposed to explain the pathogenesis of long COVID, including persistent SARS-CoV-2 reservoirs in tissues [45], immune dysregulation [18,41], alterations of the microbiota and the virome [46], reactivation of latent pathogens such as Epstein–Barr virus (EBV) and human herpesvirus 6 (HHV-6) [47], autoimmunity and immune priming through molecular mimicry [48,49], microvascular thrombosis with endothelial dysfunction [23,24,25], and abnormal vagus nerve signaling [44,48,49,50]. Nevertheless, further studies are needed to elucidate better the mechanisms underlying long COVID pathogenesis.

This comprehensive analysis highlights critical insights into the immune profiles associated with LC, emphasizing the need for targeted approaches that consider sex-specific responses in future investigations.

### 3.4. Study Limitations

This study has several limitations. We did not assess the viral reservoir or the microbiota, although our hypotheses involve viral persistence and microbiota dysregulation, including possible roles of microRNAs and virome alterations in LC mechanisms. Moreover, no other viral reservoirs were assessed, nor were tissue viral RNA or serological assays for EBV/HHV-6 carried out. Additionally, most participants were hospitalized during the acute phase, which may limit the generalizability of our findings to individuals with mild or asymptomatic infections. Long COVID status was based on the presence of self-reported symptoms, without the use of standardized functional assessments (e.g., fatigue scores, cognitive tests). Considering that many participants were hospitalized during the first wave of the pandemic, when interstitial pneumonia and respiratory failure were common, it is possible that some patients with persistent respiratory symptoms at baseline were actually experiencing pulmonary sequelae rather than long COVID syndrome. The absence of a matched healthy control group also limits the ability to distinguish COVID-19-specific immune changes from those related to other chronic conditions or the natural aging process. Finally, given the observational nature of the study, causal relationships between immune markers and long COVID cannot be established. Due to the limited sample size, a multivariable analysis to control for potential confounders was not feasible.

Our study has some strengths. It includes follow-up assessments at multiple time points (3 and 12 months post infection), allowing for the evaluation of dynamic changes in endothelial/inflammatory and immune responses over time. This analysis integrates several biomarkers, including coagulative factors, and SARS-CoV-2-specific T-cell responses, providing a comprehensive understanding of the pathophysiology of long COVID over time.

This cohort represents a real-world unvaccinated population, capturing a wide spectrum of clinical presentations in terms of symptom type and severity. Moreover, the study explores sex-specific differences in inflammatory markers (e.g., TNF-α and IL-8), and in SARS-CoV-2-specific T-cell response, contributing to the growing evidence of sex-related disparities in COVID-19 and long COVID outcomes.

Finally, our findings highlight potential biomarkers that may serve as early indicators of the development or persistence of long COVID sequelae, thereby supporting improved clinical monitoring and management of affected patients.

## 4. Materials and Methods

### 4.1. Study Design and Cohort

This is a prospective, case–control study enrolling 196 unvaccinated patients consecutively evaluated at the post-COVID clinic of the Italian National Institute for Infectious Diseases “Lazzaro Spallanzani.” Participants were enrolled between May and June 2020 and were referred either from the hospital or from community healthcare services.

Inclusion criteria were age >18 years, confirmed SARS-CoV-2 infection (positive swab ≥ 90 days before baseline), and signed informed consent. Acute SARS-CoV-2 infection was defined by the first positive RT-PCR or antigen test on a nasopharyngeal swab. Long COVID was defined as the development or persistence of symptoms attributable to SARS-CoV-2 infection, not explained by alternative diagnoses, occurring at least three months after the acute infection [51].

Symptoms were recorded by the physician during the clinical evaluation. Each patient was asked about the presence of the following symptoms: fatigue, concentration or memory deficits, reduced exercise tolerance, dyspnea, arthralgia, myalgia, dysautonomia, neuropsychiatric symptoms (e.g., insomnia, low mood, nervousness), and loss of smell or taste. Any additional symptoms reported by the patients were also documented.

Patients were assigned to the long COVID group (Ongoing LC, Ong LC) if they presented with at least one symptom at the first visit (T3M), and to the asymptomatic (Never LC, Nev LC) group if they did not. The two study cohorts were followed up for 12 months (T12M). After one year post infection, none of the enrolled patients showed symptomatology (Resolved LC, Res LC).

Blood samples (plasma and cells) were collected at three months (T3M) and twelve months (T12M) after SARS-CoV-2 infection.

Most patients (90.3%) underwent the baseline (BL) visit in June 2020. Forty-eight participants attended the twelve-month follow-up visit (T12M), while the remaining patients were lost to follow-up.

Demographic, epidemiological, laboratory and clinical data were recorded using a standardized electronic database of the National Institute for Infectious Diseases “L. Spallanzani (ReCOVeRI Study)”. The study was performed in accordance with the Declaration of Helsinki.

Laboratory assessments were conducted during hospitalization and follow-up was performed according to clinical judgment and internal protocols. Registry management followed EUnetHTA standards, as outlined in the Registry Evaluation and Quality Standards Tool in 2019.

Blood samples (plasma and cells) were collected at three months (T3M) and twelve months (T12M) after SARS-CoV-2 infection. All participants provided informed consent for the collection and use of their data for research purposes.

### 4.2. Assessment of Coagulation and Inflammation Factors

Plasma samples were obtained after centrifugation of whole blood for 10 min at 1800 rpm and immediately stored at −80 °C until analysis. Plasma levels of D-dimer, E-Sel, ICAM-1, VCAM-1, IL-1β, IL-6, IL-8, and TNF-α were quantified using an automated ELISA assay (ELLA microfluidic analyzer, ProteinSimple, Bio-Techne, Minneapolis, MN, USA).

### 4.3. Peripheral Lymphocyte Isolation and Quantification

Peripheral blood mononuclear cells (PBMCs) were isolated by density gradient centrifugation using Lympholyte (Cedarlane, Burlington, ON, Canada), counted with Trypan Blue exclusion, and cryopreserved in Fetal Bovine Serum (FBS; Euroclone, Milan, Italy) containing 10% DMSO (Euroclone, Milan, Italy) under liquid nitrogen vapors.

For experiments, PBMCs were thawed in complete RPMI-1640 medium supplemented with 10% FBS, 2 mM glutamine, 50 IU/mL penicillin, and 50 µg/mL streptomycin (Corning, NY, USA). After a single wash, cells were resuspended at a concentration of 1 × 10^6^ cells/mL, counted by Trypan Blue exclusion, and immediately used for downstream assays.

### 4.4. SARS-CoV-2-Specific T-Cell Response

The SARS-CoV-2-specific T-cell response was evaluated by measuring interferon-γ (IFN-γ) secretion using a standard ELISpot assay. Briefly, PBMCs (3 × 10^5^ cells/well) were seeded into ELISpot plates (AID) and stimulated with spike protein peptide pools (0.1 µg/mL; PepTivator SARS-CoV-2 Prot_S1, Prot_S, and Prot_S+, Miltenyi Biotec, Bologna, Italy) or nucleocapsid protein (1 µg/mL; PepTivator SARS-CoV-2 Prot_N, Miltenyi Biotec) for 20 h at 37 °C in 5% CO_2_. Stimulation with the T-cell superantigen SEB (200 nM; Sigma-Merck, Darmstadt, Germany) served as a positive control. Following incubation, the assay was developed according to the manufacturer’s protocol, and spot-forming cells (SFCs) were quantified using the AID ELISpot Reader (version 7.0). Results were expressed as SFCs per 10^6^ PBMCs, after subtraction of background values.

### 4.5. Statistical Analysis

Non-parametric Mann–Whitney and Wilcoxon tests were used to assess differences in quantitative variables between hospitalized and non-hospitalized patients, asymptomatic and symptomatic persons, and for longitudinal comparisons within LC subjects, respectively. A heat map analysis was performed for all markers at both time points, and the results of the Spearman correlation tests are reported. Statistical analyses were performed using GraphPad Prism software version 8.0 (GraphPad Software, San Diego, CA, USA).

## 5. Conclusions

In summary, our data highlight a complex and dynamic interplay among coagulative, endothelial, inflammatory, and immune pathways in post-COVID individuals, independent of the presence of symptoms. These interactions evolve and are influenced by patient age, with evidence of persistent immune activation and vascular stress up to one year after infection. In addition, inflammatory mechanisms appear to differ between sexes, with a more critical endothelial activation in males and a worse T-specific spike response as well as a higher TNF-activation in females [52]. As demonstrated in previous studies, our findings show that most cytokine abnormalities are not associated with the presence of symptoms. This highlights the need to identify new biomarkers (e.g., microRNAs, markers of mitochondrial damage, autoantibodies against G protein-coupled receptors) that are sex-specific and, ideally, personalized.

### Future Directions

Future studies should focus on host predisposing factors to understand why only some patients with immuno-inflammatory abnormalities develop the disease. In particular, it will be essential to investigate the sensitivity of certain receptors to the involved biomarkers, in addition to quantifying these markers and exploring new, as yet unidentified, mechanisms of cellular damage. The tip of the iceberg has been identified, but its main body still lies beneath the surface. Our findings have important implications, adding a piece to the puzzle of understanding the pathophysiology of long COVID and guiding future monitoring and therapeutic strategies.

## Figures and Tables

**Table 1:** Demographic and clinical characteristics at first post-COVID follow-up visit.

Characteristic	N (%)/Median (IQR)
Total subjects	196
Sex	
Male	119 (60.7)
Female	77 (39.3)
Age at visit (years)	56.5 (49.5–64.9)
Date of first visit	
May 2020	19 (9.7)
June 2020	177 (90.3)
(Clinic began in late May 2020)	
Time from first positive test to visit (days)	86 (78–91)
Previous COVID-19 hospitalization (at the institute)	154 (78.6)
Time from discharge to visit (days)	82 (76–89)
Length of stay (days)	11 (8–18)
COVID-19 hospitalization (any facility)	
No	127 (64.8)
Yes	14 (7.1)
Missing	55 (28.1)
Symptomatic at visit	66 (33.7)
1 symptom	45 (68.2 of symptomatic)
2+ symptoms	21 (31.8)
2 symptoms	14
3 symptoms	5
4 symptoms	2
Total number of symptoms reported	96
Most frequent symptoms	
Dyspnea/cough/shortness of breath	36 (54.5 of symptomatic)
Fatigue	20 (30.3)
Other symptoms	16 (24.2)
Neuropsychological symptoms	9 (13.6)
Anosmia/ageusia/dysosmia/dysgeusia	8 (12.1)
Arthromyalgias	7 (10.6)

## Data Availability

Demographic, epidemiological, laboratory, and clinical data were recorded using a standardized electronic database of the National Institute for Infectious Diseases “L. Spallanzani (ReCOVeRI Study)”. Data are available upon reasonable request.

## Supplementary Materials

The following supporting information can be downloaded at: https://www.mdpi.com/article/10.3390/ijms262110412/s1.

## References

[1] Jun-Won S., Kim S.E., Kim Y., Kim E.J., Kim T., Kim T., Lee S.O., Lee E., Lee J., Seo Y.B. (2024). Updated Clinical Practice Guidelines for the Diagnosis and Management of Long COVID. Infect. Chemother..

[2] O’Mahoney L.L., Routen A., Gillies C., Ekezie W., Welford A., Zhang A., Karamchandani U., Simms-Williams N., Cassambai S., Ardavani A. (2022). The prevalence and long-term health effects of Long COVID among hospitalised and non-hospitalised populations: A systematic review and meta-analysis. EClinicalMedicine.

[3] Bohmwald K., Diethelm-Varela B., Rodríguez-Guilarte L., Rivera T., Riedel C.A., González P.A., Kalergis A.M. (2024). Pathophysiological, immunological, and inflammatory features of long COVID: Review. Front. Immunol..

[4] Asadi-Pooya A.A., Akbari A., Emami A., Lotfi M., Rostamihosseinkhani M., Nemati H., Barzegar Z., Kabiri M., Zeraatpisheh Z., Farjoud-Kouhanjani M. (2021). Risk factors associated with long COVID syndrome: A retrospective study. Iran. J. Med. Sci..

[5] Augustin M., Schommers P., Stecher M., Dewald F., Gieselmann L., Gruell H., Horn C., Vanshylla K., Di Cristanziano V., Osebold L. (2021). Post-COVID syndrome in non-hospitalized patients with COVID-19: A longitudinal prospective cohort study. Lancet Reg. Health Eur..

[6] Aul D.R., Gates D.J., Draper D.A., Dunleavy A., Ruickbie S., Meredith H., Walters N., van Zeller C., Taylor V., Bridgett M. (2021). Complications after discharge with COVID-19 infection and risk factors associated with development of post-COVID pulmonary fibrosis. Respir. Med..

[7] Aydin S., Unver E., Karavas E., Yalcin S., Kantarci M. (2021). Computed tomography at every step: Long coronavirus disease. Respir. Investig..

[8] Ayoubkhani D., Bermingham C., Pouwels K.B., Glickman M., Nafilya V., Zaccardi F., Khunti K., Alwan N.A., Walker S. (2022). Trajectory of long COVID symptoms after COVID-19 vaccination: Community-based cohort study. BMJ.

[9] Wu X., Xiang M., Jing H., Wang C., Novakovic V.A., Shi J. (2024). Damage to endothelial barriers and its contribution to long COVID. Angiogenesis.

[10] Fogarty H., Townsend L., Morrin H., Ahmad A., Comerford C., Karampini E., Englert H., Byrne M., Bergin C., O’Sullivan J.O. (2021). Persistent endotheliopathy in the pathogenesis of Long COVID syndrome. J. Thromb. Haemost..

[11] Fan B.E., Wong S.W., Sum C.L.L., Lim G.H., Leung B.P., Tan C.W., Ramanathan K., Dalan R., Cheung C., Lim X.R. (2022). Hypercoagulability, endotheliopathy, and inflammation approximating 1 year after recovery: Assessing the long-term outcomes in COVID-19 patients. Am. J. Hematol..

[12] Boccatonda A., Campello E., Simion C., Simioni P. (2023). Long-term hypercoagulability, endotheliopathy and inflammation following acute SARS-CoV-2 infection. Expert Rev. Hematol..

[13] Chioh F.W., Fong S.W., Young B.E., Wu K.X., Siau A., Krishnan S., Chan Y.-H., Carissimo G., Ly Teo L., Gao F. (2021). Convalescent COVID-19 patients are susceptible to endothelial dysfunction due to persistent immune activation. eLife.

[14] Zanoli L., Gaudio A., Mikhailidis D.P., Katsiki N., Castellino N., Lo Cicero L., Geraci G., Sessa C., Fiorito L., Marino F. (2022). Vascular dysfunction of COVID-19 is partially reverted in the long-term. Circ. Res..

[15] Lambadiari V., Mitrakou A., Kountouri A., Thymis J., Katogiannis K., Korakas E., Varlamos C., Andreadou I., Tsoumani M., Triantafyllidi H. (2021). Association of COVID-19 with impaired endothelial glycocalyx, vascular function and myocardial deformation 4 months after infection. Eur. J. Heart Fail..

[16] Renner K., Stauffenberg F., Paulus M., Neumayer S., Winter-Köhler F., Buchtler S., Schmalenberger D., Blaas S., Mohr A., Pfeifer M. (2025). Hyper-reactivity of CD8+ T cells and high expression of IL-3 correlates with occurrence and severity of Long-COVID. Clin. Immunol..

[17] Schulthei C., Willscher E., Paschold L., Gottschick C., Klee B., Henkes S.-S., Bosurgi L., Dutzmann J., Sedding D., Frese T. (2022). The IL-1beta, IL-6, and TNF-alpha cytokine triad is associated with post-acute sequelae of COVID-19. Cell Rep. Med..

[18] Phetsouphanh C., Darley D.R., Wilson D.B., Howe A., Mee Ling Munier C., Patel S.K., Juno J.A., Burrell L.M., Kent S.J., Dore G.J. (2022). Immunological dysfunction persists for 8 months following initial mild-to-moderate SARS-CoV-2 infection. Nat. Immunol..

[19] Peluso M.J., Lu S., Tang A.F., Durstenfeld M.S., Ho H.E., Goldberg S.A., Forman C.A., Munter S.E., Hoh R., Tai V. (2021). Markers of Immune Activation and Inflammation in Individuals With Postacute Sequelae of Severe Acute Respiratory Syndrome Coronavirus 2 Infection. J. Infect. Dis..

[20] Alfaro E., Díaz-García E., García-Tovar S., Galera R., Casitas R., Torres-Vargas M., López-Fernández C., Añón J.M., García-Río F., Cubillos-Zapata C. (2024). Endothelial dysfunction and persistent inflammation in severe post-COVID-19 patients: Implications for gas exchange. BMC Med..

[21] Conti P., Caraffa A., Gallenga C.E., Ross R., Kritas S.K., Frydas I., Younes A., Ronconi G. (2020). Coronavirus-19 (SARS-CoV-2) induces acute severe lung inflammation via IL-1 causing cytokine storm in COVID-19: A promising inhibitory strategy. J. Biol. Regul. Homeost. Agents.

[22] Pavoni V., Gianesello L., Pazzi M., Stera C., Meconi T., Covani Frigieri F. (2020). Evaluation of coagulation function by rotation thromboelastometry in critically ill patients with severe COVID-19 pneumonia. J. Thromb. Thrombolysis.

[23] Bikdeli B., Madhavan M.V., Gupta A., Jimenez D., Burton J.R., Der Nigoghossian C., Chuich T., Nabavi Nouri S., Dreyfus I., Driggin E. (2020). Pharmacological Agents Targeting Thromboinflammation in COVID-19: Review and Implications for Future Research. Thromb. Haemost..

[24] Barrett T.J., Lee A.H., Xia Y., Lin H.L., Black M., Cotzia P., Hochman J., Berger J.S. (2020). Platelet and Vascular Biomarkers Associate With Thrombosis and Death in Coronavirus Disease. Circ. Res..

[25] Ackermann M., Verleden S.E., Kuehnel M., Haverich A., Welte T., Laenger F., Vanstapel A., Werlein C., Stark H., Tzankov A. (2020). Pulmonary Vascular Endothelialitis, Thrombosis, and Angiogenesis in COVID-19. N. Engl. J. Med..

[26] Connors J.M., Levy J.H. (2020). COVID-19 and its implications for thrombosis and anticoagulation. Blood.

[27] Al-Samkari H., Karp L.R.S., Dzik W.H., Carlson J.C.T., Fogerty A.E., Waheed A., Goodarzi K., Bendapudi P.K., Bornikova L., Gupta S. (2020). COVID-19 and coagulation: Bleeding and thrombotic manifestations of SARS-CoV-2 infection. Blood.

[28] Lehmann A., Prosch H., Zehetmayer S., Gysan M.R., Bernitzky D., Vonbank K., Idzko M., Gompelmann D. (2021). Impact of persistent D-dimer elevation following recovery from COVID-19. PLoS ONE.

[29] Fong S.W., Goh Y.S., Torres-Ruesta A., Chang Z.W., Chan Y.-H., Kexin Neo V., Lee B., Duan K., Naqiah Amrun S., Yeo N.K.-W. (2023). Prolonged inflammation in patients hospitalized for coronavirus disease 2019 (COVID-19) resolves 2 years after infection. J. Med. Virol..

[30] Ruhl L., Pink I., Kühne J.F., Beushausen K., Keil J., Christoph S., Sauer A., Boblitz L., Schmidt J., David S. (2021). Endothelial dysfunction contributes to severe COVID-19 in combination with dysregulated lymphocyte responses and cytokine networks. Signal Transduct. Target. Ther..

[31] Sette A., Crotty S. (2021). Adaptive immunity to SARS-CoV and COVID-19. Cell.

[32] Tan A.T., Linster M., Tan C.W., Le Bert N., Chia W.N., Kunasegaran K., Zhuang Y., Tham C.Y.L., Chia A., Smith G.J.I. (2021). Early induction of functional SARS-CoV-specific T cells Associates with rapid viral clearance and mild disease in COVID-19 patients. Cell Rep..

[33] Littlefield K.M., Watson R.O., Schneider J.M., Neff C.P., Yamada E., Zhang M., Campbell T.B., Falta M.T., Jolley S.E., Fontenot A.P. (2022). SARS-CoV-specific T cells associate with inflammation and reduced lung function in pulmonary post-acute Sequalae of SARS-CoV. PLoS Pathog..

[34] Wiech M., Chroscicki P., Swatler J., Stepnik D., De Biasi S., Hampel M., Brewinska-Olchowik M., Maliszewska A., Sklinda K., Durlik M. (2022). Remodeling of T cell Dynamics during long COVID is dependent on severity of SARS-CoV infection. Front. Immunol..

[35] Peluso M.J., Deitchman A.N., Torres L., Iyer N.S., Munter S.E., Nixon C.C., Donatelli J., Thanh C., Takahashi S., Hakim J. (2021). Long-term SARS-CoV-2-specific immune and inflammatory responses in individuals recovering from COVID-19 with and without post-acute symptoms. Cell Rep..

[36] Sjöwall J., Hjorth M., Gustafsson A., Göransson R., Larsson M., Waller H., Nordgren J., Nilsdotter-Augustinsson A., Nyström S. (2022). SARS-CoV-2 Specific Antibody Response and T Cell-Immunity in Immunocompromised Patients up to Six Months Post COVID: A Pilot Study. J. Clin. Med..

[37] Davis H.E., McCorkell L., Vogel J.M., Topol E.J. (2023). Author correction: Long COVID: Major findings, mechanisms and recommendations. Nat. Rev. Microbiol..

[38] Palladini M., Mazza M.G., Bravi B., Bessi M., Lorenzi M.C., Spadini S., De Lorenzo R., Rovere-Querini P., Furlan R., Benedetti F. (2025). Sex-Specific Inflammatory Profiles Affect Neuropsychiatric Issues in COVID-19 Survivors. Biomolecules.

[39] Ober V., Völk F., Sbierski-Kind J., Grüner E., Stirner R., Reiling G., Feldmann S., Ibarra G., Seybold U., Stubbe H. (2025). Immune disturbances in individuals with post-COVID syndrome are not characterized by enhanced SARS-CoV--specific immunity. J. Infect. Dis..

[40] Martínez-Fleta P., Marcos M.C., Jimenez-Carretero D., Galván-Román J.M., Girón-Moreno R.M., Calero-García A.A., Arcos-García A., Martín-Gayo E., de la Fuente H., Esparcia-Pinedo L. (2024). Imbalance of SARS-CoV--specific CCR6+ and CXCR3 + CD4 + T cells and IFN-gamma + CD8+ T cells in patients with Long-COVID. Clin. Immunol..

[41] Yin K., Peluso M.J., Luo X., Thomas R., Shin M.G., Neidleman J., Andrew A., Young K.C., Ma T., Hoh R. (2024). Long COVID manifests with T cell dysregulation, inflammation and an uncoordinated adaptive immune response to SARS-CoV-2. Nat. Immunol..

[42] Guerrera G., Sambucci M., Timperi E., Picozza M., Misiti A., Placido R., Corbisiero S., D’Orso S., Termine A., Fabrizio C. (2025). Identification of an immunological signature of long COVID syndrome. Front. Immunol..

[43] Bai F., Tomasoni D., Falcinella C., Barbanotti D., Castoldi R., Mulè G., Augello M., Mondatore D., Allegrini M., Cona A. (2022). Female gender is associated with long COVID syndrome: A prospective cohort study. Clin. Microbiol. Infect..

[44] Camici M., Del Duca G., Brita A.C., Antinori A. (2024). Connecting dots of long COVID-19 pathogenesis: A vagus nerve- hypothalamic-pituitary- adrenal-mitochondrial axis dysfunction. Front. Cell. Infect. Microbiol..

[45] Proal A.D., VanElzakker M.B. (2021). Long COVID or post-acute sequelae of COVID-19 (PASC): An overview of biological factors that may contribute to persistent symptoms. Front. Microbiol..

[46] de Almeida V.M. (2022). Gut microbiota from patients with mild COVID-19 cause alterations in mice that resemble post-COVID syndrome. Res. Sq..

[47] Su Y., Yuan D., Chen D.G., Ng R.H., Wang K., Choi J., Li S., Hong S., Zhang R., Xie J. (2022). Multiple early factors anticipate post-acute COVID-19 sequelae. Cell.

[48] Wallukat G., Hohberger B., Wenzel K., Fürst J., Schulze-Rothe S., Wallukat A., Hönicke A.-S., Müller J. (2021). Functional autoantibodies against G-protein coupled receptors in patients with persistent long-COVID-19 symptoms. J. Transl. Autoimmun..

[49] Arthur J.M., Forrest J.C., Boehme K.W., Kennedy J.L., Owens S., Herzog C., Liu J., Harville T.O. (2021). Development of ACE2 autoantibodies after SARS-CoV-2 infection. PLoS ONE.

[50] Spudich S., Nath A. (2022). Nervous system consequences of COVID-19. Science.

[51] https://www.who.int/europe/news-room/fact-sheets/item/post-COVID-19-condition.

[52] Ganesh R., Yadav S., Hurt R.T., Mueller M.R., Aakre C.A., Gilman E.A., Grach S.L., Overgaard J., Snyder M.R., Collins N.M. (2024). Pro Inflammatory Cytokines Profiles of Patients With Long COVID Differ Between Variant Epochs. J. Prim. Care Community Health.

