# Supplementary material for: Inflammatory Milieu and Specific T-Cell Response Observed Three Months and One Year After SARS-CoV-2 Infection in Long COVID Subjects
Source: Int J Mol Sci. 2025 Oct 27;26(21):10412. doi: 10.3390/ijms262110412 (PMC12607545; doi:10.3390/ijms262110412)
Supplement: Supplementary file 1 [file ijms-26-10412-s001.zip › ijms-3917684-supplementary.pdf]

Supplementary data

Inflammatory milieu and specific T-cell response observed three months and one year after SARS-CoV-2 infection in Long COVID subjects

Eleonora Cimini[1§], Alessandra Vergori[2§], Claudia Cimaglia[3], Eleonora Tartaglia[4], Marta Camici[2*], Stefania Notari[1], Francesca Colavita[4], Giulia Matusali[4], Ilaria Mastrorosa[2], Valentina Mazzotta[2], Pierangelo Chinello[5], Paola Mencarini[5], Maria Letizia Giancola[5], Amina Abdeddaim[5], Rita Casetti[1], Germana Grassi[1], Simona Gili[1], Flavia Cristofanelli[1], Fabrizio Maggi[4], Pierluca Piselli[3], Enrico Girardi[6], Andrea Antinori[2].

Supplementary Figure 1. Coagulative and endothelial factors after three- and twelve-months post SARS-CoV-2 infection in LC hospitalized and non-hospitalized subjects. Plasmatic concentrations of D-Dmer, E-Sel, ICAM-1, and VCAM-1 were measured in 196 LC subjects at three- and twelve-month post SARS-CoV-2 infection by the ELISA test.

Supplementary Figure 2. Inflammatory factors after three- and twelve-months post SARS-CoV-2 infection in LC hospitalized and non-hospitalized subjects. Plasmatic concentrations of inflammatory factors such as IL-6, IL-1, IL-8, and TNF-α were measured in 196 LC subjects at three- and twelve-month post SARS-CoV-2 infection by the ELISA test.

Supplementary Figure 3. T cell response to SARS-CoV-2 after three- and twelve-months post-infection in LC hospitalized and non-hospitalized subjects. Spike and Nucleocapsid specific T cell responses were measured in PBMC from 196 LC subjects at three- and twelve-months post SARS-CoV-2 infection by Elispot assay.

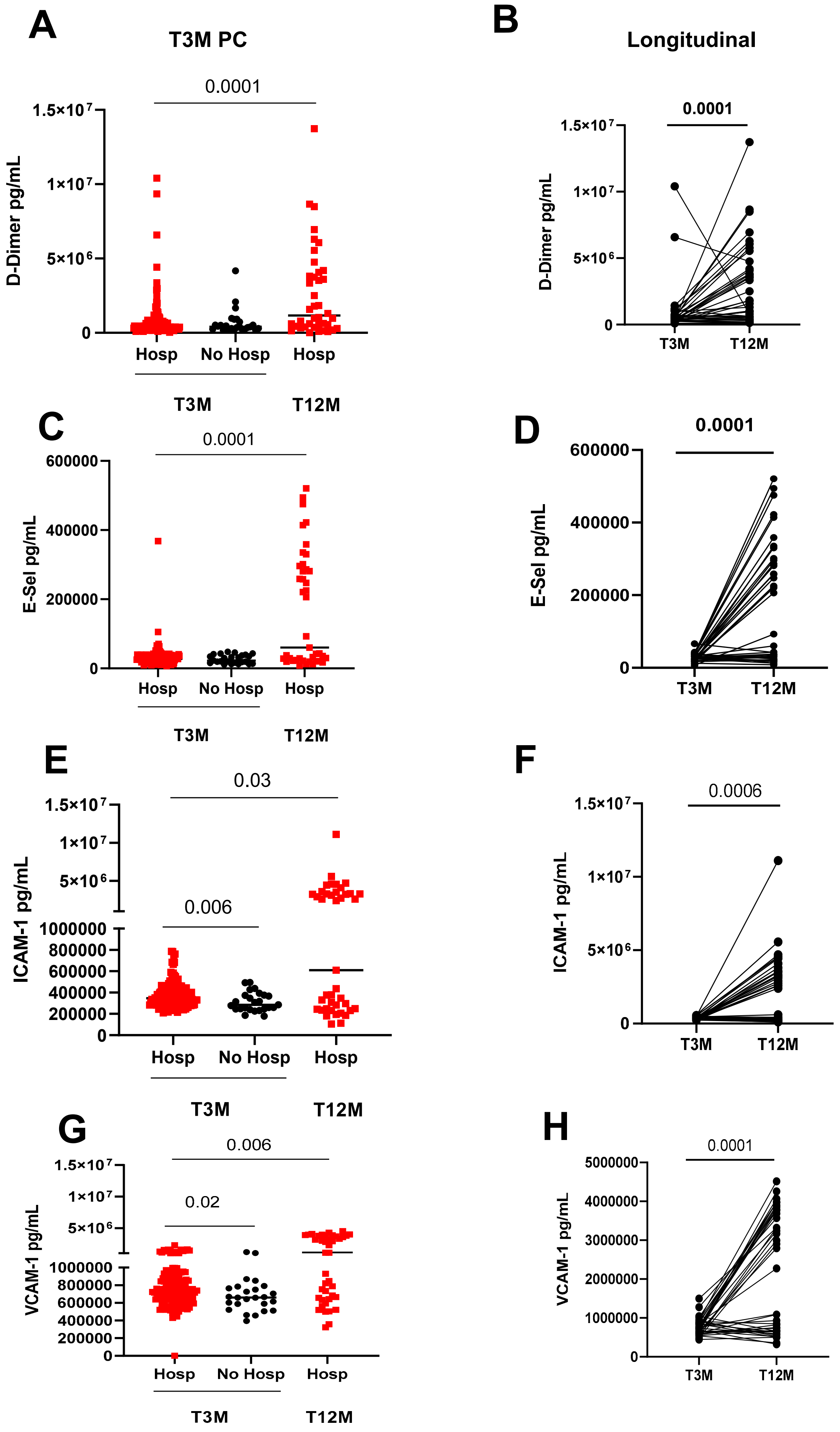

Supplementary Figure 1

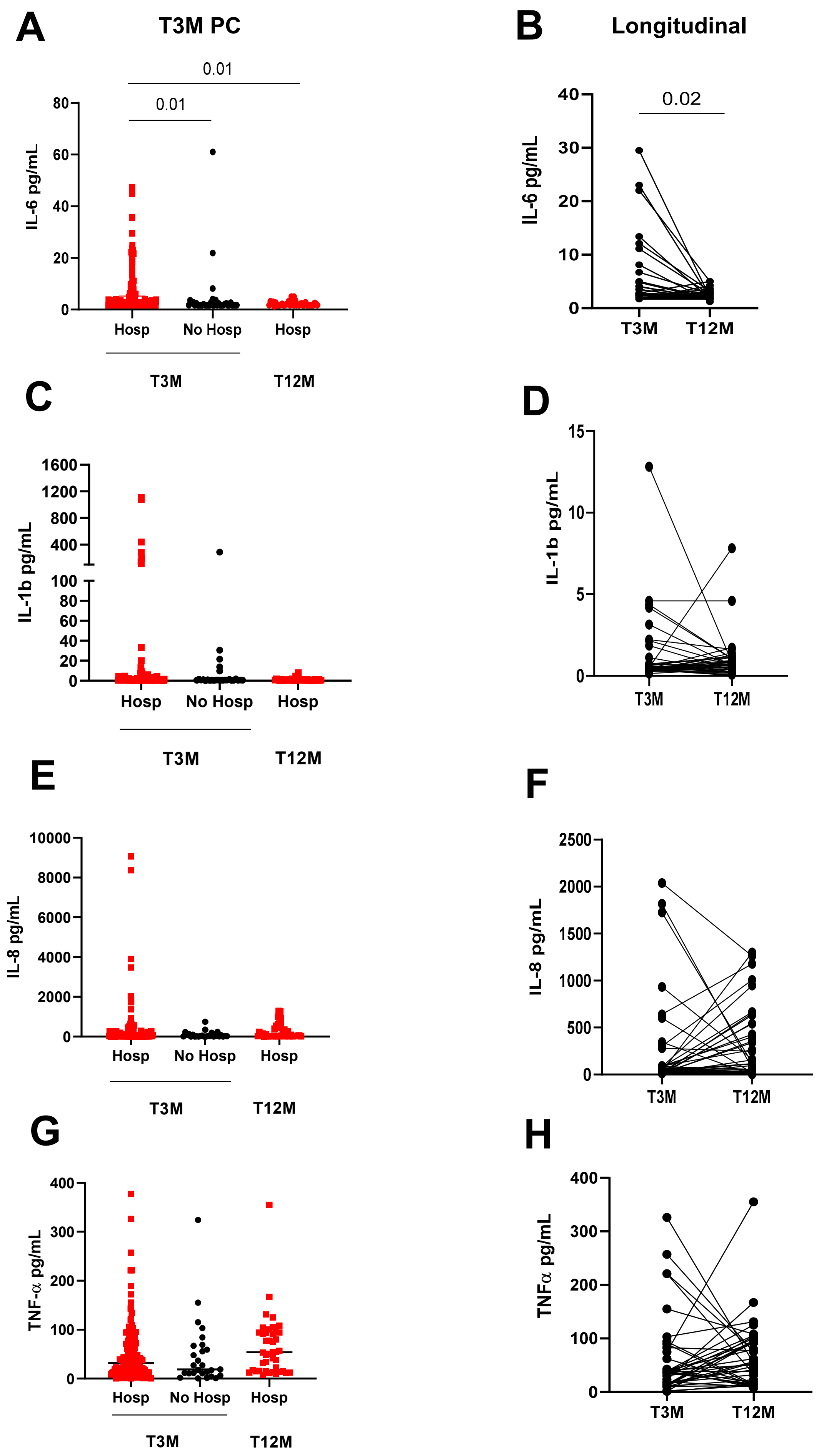

Supplementary Figure 2

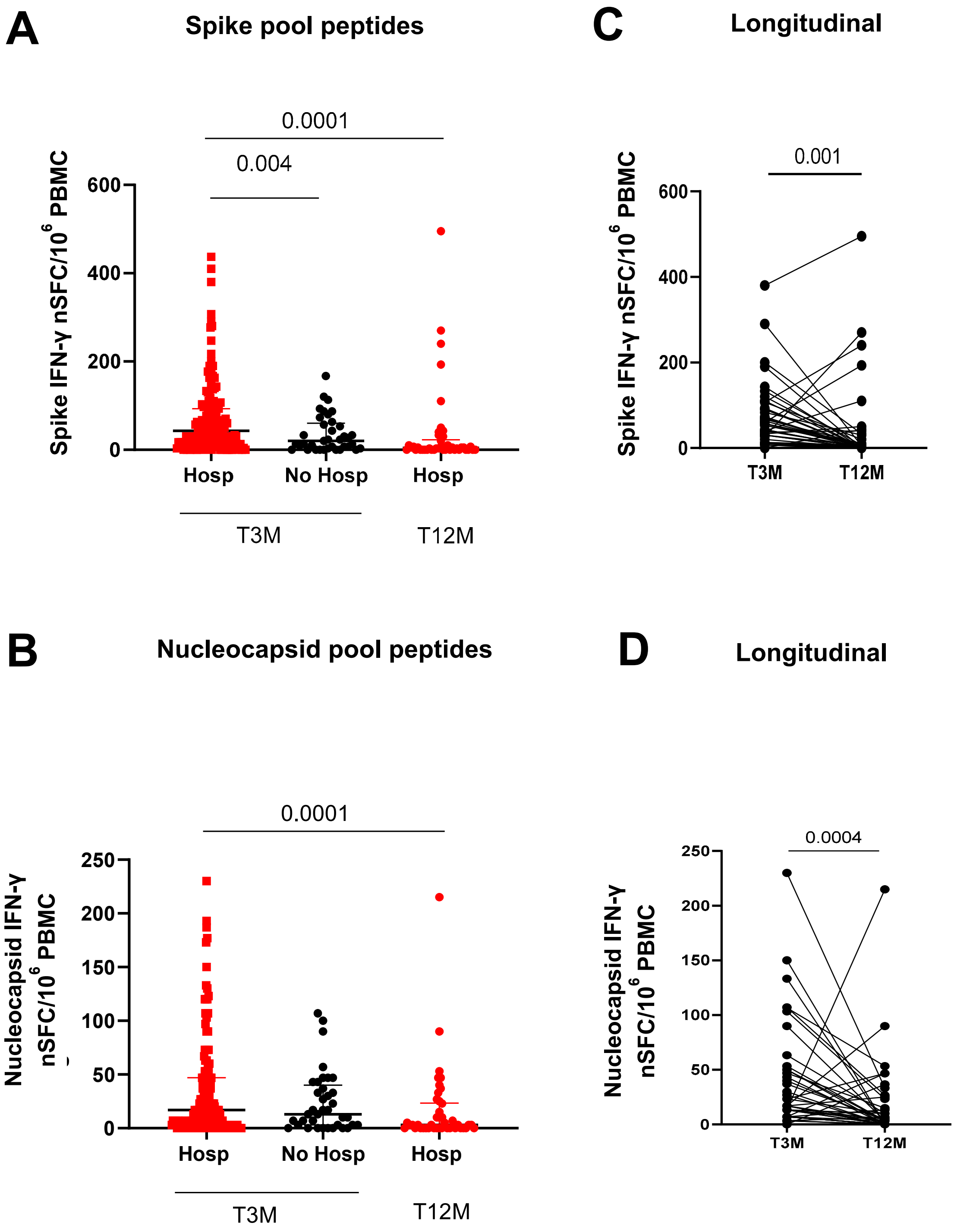

Supplementary Figure 3
